# Toxicity of *meta*-Tyrosine

**DOI:** 10.3390/plants10122800

**Published:** 2021-12-17

**Authors:** Marcin Tyminski, Katarzyna Ciacka, Pawel Staszek, Agnieszka Gniazdowska, Urszula Krasuska

**Affiliations:** Department of Plant Physiology, Institute of Biology, Warsaw University of Life Sciences, Nowoursynowska 159, 02-776 Warsaw, Poland; katarzyna_ciacka@sggw.edu.pl (K.C.); pawel_staszek1@sggw.edu.pl (P.S.); agnieszka_gniazdowska@sggw.edu.pl (A.G.); urszula_krasuska@sggw.edu.pl (U.K.)

**Keywords:** allelochemical, non-proteinogenic amino acids, reactive oxygen species, cancer, fescue

## Abstract

L-Tyrosine (Tyr) is one of the twenty proteinogenic amino acids and also acts as a precursor for secondary metabolites. Tyr is prone to modifications, especially under conditions of cellular redox imbalance. The oxidation of Tyr precursor phenylalanine leads to the formation of Tyr non-proteinogenic isomers, including *meta*-Tyr (*m*-Tyr), a marker of oxidative stress. The aim of this review is to summarize the current knowledge on *m*-Tyr toxicity. The direct *m*-Tyr mode of action is linked to its incorporation into proteins, resulting in their improper conformation. Furthermore, *m*-Tyr produced by some plants as an allelochemical impacts the growth and development of neighboring organisms. In plants, the direct harmful effect of *m*-Tyr is due to its modification of the proteins structure, whereas its indirect action is linked to the disruption of reactive oxygen and nitrogen species metabolism. In humans, the elevated concentration of *m*-Tyr is characteristic of various diseases and ageing. Indeed, *m*-Tyr is believed to play an important role in cancer physiology. Thus, since, in animal cells, *m*-Tyr is formed directly in response to oxidative stress, whereas, in plants, *m*-Tyr is also synthesized enzymatically and serves as a chemical weapon in plant–plant competition, the general concept of *m*-Tyr role in living organisms should be specified.

## 1. Introduction

Twenty canonical amino acids (AAs) are the base of the proteins structure in living organisms. Besides them, numerous non-proteinogenic amino acids (NPAAs) are produced in plants [1]. Some of the NPAAs are described as antimetabolites, analogs of proteinogenic AAs, and serve as toxins. The negative effect of a specific NPAA may be removed by the application of its proteinogenic analog.

Tyrosine (Tyr-α-amino-β-(*p*-hydroxyphenyl)propionic acid) belongs to the group of aromatic amino acids (AAAs), and occurs as two optical isomers (enantiomers) L-Tyr and D-Tyr [2]. Most living organisms utilize L-Tyr to form proteins/peptides. The content of this AA in Arabidopsis (*Arabidopsis thaliana* (L.) Henh.) leaves is about 5 pmol mg^−1^ fresh weight (FW) ([3] and citations herein). Tyr is a Phe derivative, with a hydroxyl group located in the *para*- position on the benzyl ring (*p*-Tyr) [4]. In proteins, Tyr residues are often located on the surface and are prone to various post-translational modifications, such as phosphorylation or nitration [5]. In plants, AAAs are precursors for many secondary metabolites belonging to, e.g., groups of flavonoids, tannins or lignins [6], and alkaloids [6,7]. Although the main precursor for lignins biosynthesis is Phe, recent investigations on stiff brome (*Brachypodium distachyon* (L.) P.Beauv.) revealed that the synthesis of these compounds directly from Tyr is also possible [8,9]. As Tyr is implicated in the plastoquinones biosynthesis, its deficiency may disturb the electron transport chain in photosystems [4,10]. In plants, the increased concentration of Tyr is observed during the reaction against biotic stresses. The Tyr content in shimbillo (*Inga umbellifera* (Vahl) Steud. ex DC) expanding leaves is about 6.6 to 10% of the dry weight (DW), although, in mature leaves, it decreases to only 0.05% of the DW [11]. Herbivore attacks on this tropical legume tree increased the Tyr concentration in the leaves [12]. In response to herbivores, Coley et al. [13] correlated the Tyr accumulation in the young leaves of genus *Inga* to the overexpression of the genes encoding the enzymes of Tyr biosynthesis.

In sorgo (*Sorghum bicolor* L. Moench.), Tyr is a precursor in the synthesis of dhurrin, a cyanogenic glucoside, which has a defensive role against herbivores [14]. The toxicity of Tyr (mixture of L-Tyr and D-Tyr isomers) at elevated concentrations was confirmed for insects [11]. The adddition of the Tyr racemate (at the level of 4%) into the diet of the tobacco budworm (*Heliothis virescens*) completely inhibited larvae growth [11]. Ten times higher than the physiological concentration of L-Tyr in plasma led to severe brain damage and DNA damage in Wistar rats (*Rattus norvegicus domestica*), possibly because of an oxidative stress [15]. Such a toxic concentration of L-Tyr is observed in patients suffering from tyrosinemia type II [15].

**Figure 1:**
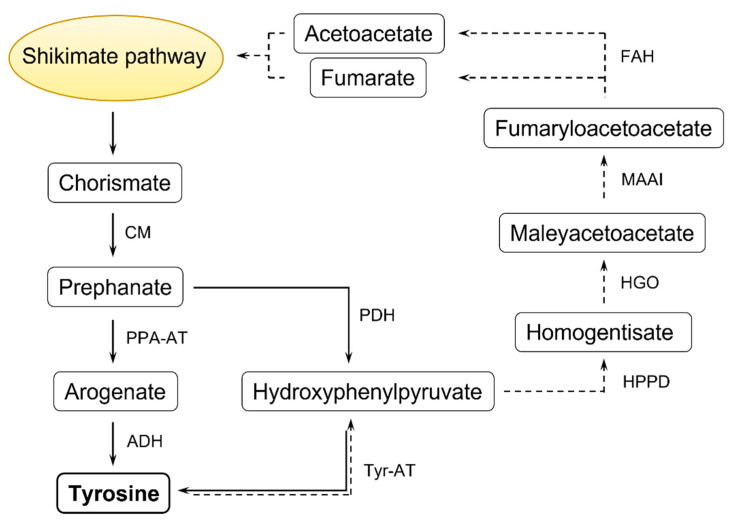
Tyr synthesis (solid line) and Tyr catabolism (dashed line). Enzymes: chorismate mutase (CM), prephenate aminotransferase (PPA-AT), arogenate dehydrogenase (ADH), prephenate dehydrogenase (PDH), Tyr aminotransferase (Tyr-AT), 4-hydroxyphenylpyruvate dioxygenase (HPPD), homogentisate 1,2-dioxygenase (HGO), maleylacetoacetate isomerase (MAAI), fumarylacetoacetate hydrolase (FAH). Substrates or products: *p*-hydroxyphenylpyruvate (HPP), homogentisate (HGA). According to [4] with some modifications.

The *de novo* synthesis of Tyr occurs only in plants and microbes. Chorismate, the final product of the shikimate pathway, is a precursor for Phe and Tyr biosynthesis in plants, bacteria, and fungi ([16] and references herein). In plants, chorismate mutase (CM) converts chorismate into prephenate, the main substrate for these AAs’ biosynthesis (Figure 1). Then, prephenate may be quickly transformed into arogenate in the reaction catalyzed by prephenate aminotransferase (PPA-AT), and further to Tyr by arogenate dehydrogenase (ADH). Another possible pathway of Tyr biosynthesis is the alteration of prephenate, in the presence of nicotinamide adenine dinucleotide phosphate (NADP^+^), in the reaction catalyzed by prephenate dehydrogenase (PDH). The product, *p*-hydroxyphenylpyruvate (HPP), undergoes transamination into Tyr in the reaction catalyzed by Tyr aminotransferase (Tyr-AT) [4]. In plants, Phe and Tyr are synthesized mostly in plastids, and most likely exported to the cytosol via plastid located AAA-transporters [7,17]. Animals obtain Tyr from the diet; it has been demonstrated that Phe may be converted to *p*-Tyr in the reaction catalyzed by Phe hydroxylase [18].

The catabolism of Tyr is a multi-step process occurring mostly under stress conditions and senescence. It is suggested that it takes place in the cytosol ([3] and citations herein). The degradation of Tyr starts through its transamination into HPP by Tyr-AT (Figure 1). Next, the 4-hydroxyphenylpyruvate dioxygenase (HPPD) converts HPP to homogentisate (HGA), a precursor for plastoquinones or vitamin E [19]. The formation of HGA in the AAs’ degradation pathway is followed by the conversion into maleylacetoacetate by homogentisate 1,2-dioxygenase (HGO). The role of maleylacetoacetate isomerase (MAAI) is to produce fumarylacetoacetate—the next intermediate of Tyr catabolism. The degradation of Tyr in the oxidative pathway ends with the release of the acetoacetate and fumarate from fumarylacetoacetate in the reaction catalyzed by fumarylacetoacetate hydrolase (FAH) [4]. Further, these compounds can be reintegrated into the shikimate pathway (Figure 1).

## 2. Tyrosine Structural Isomers: *meta*-, *ortho*-, *para*-Tyr

**Figure 2:**
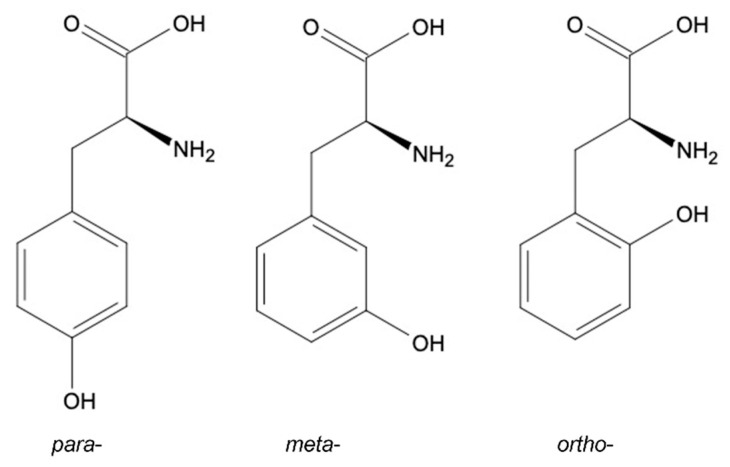
Structural isomers of Tyr: *para*-Tyr, *meta*-Tyr, *ortho*-Tyr.

Depending on the location of a hydroxyl group in the benzyl ring, three structural Tyr isomers are described: (i) *para*-(*p*-Tyr), which is the most common product of metabolic reactions integrated into proteins; and (ii) *meta*-(*m*-Tyr) and (iii) *ortho*-(*o*-Tyr), which are both products of the oxidation of Phe, known also as markers of oxidative stress [18] (Figure 2). Although the *o*-Tyr has been proven to have a deteriorative effect in animal cells [20], its toxicity in plants is still not fully described.

Various biotic and abiotic stressors lead to the induction of oxidative stress in plants [21]. Oxidative stress is an imbalance between oxidants and antioxidants in favor of the oxidants, leading to a disruption of redox signaling and molecular damage [22]. Under the conditions of ROS over-accumulation, *m*-Tyr and *o*-Tyr may be formed non-enzymatically via Phe hydroxylation or oxidation. The hydroxyl radical attack on the Phe ring initiates a two-step process, producing the highly reactive hydroxyphenylalanine radical intermediate. The formation of a stable isomer from the intermediate terminates the reaction. The mechanism of the stable end product generation may be the result of one of three possible mechanisms: abstraction, oxygenation, or disproportionation ([18] and citations herein). Tyr non-proteinogenic isomers can also emerge during the exposure of Phe to highly energetic radiation or in the reaction with peroxynitrite (ONOO^−^), a compound formed from the superoxide anion (O^2−^) and nitric oxide (NO). The hydroxylation of Phe in the presence of ONOO^−^ occurs partially via hydroxyl radicals formation [23].

## 3. Fescue as the Biological Source of *m*-Tyr

The genus of fescue (*Festuca* L.) includes about 450 species around the world [24]. Of agronomical importance are mainly fine leaf fescues (*Festuca rubra* L. spp. rubra, *Festuca rubra* L. spp. trichophylla Gaud., and spp. littoralis (Meyer) Auquiz), fescues forming clumps (*Festuca rubra* L. spp. commutata Gaud.), and sheep fescue (*Festuca ovina* L.) [25]. Fine leaf fescues were used as golf grasslands in the 16th century and are still typical for golf courses, sports fields, and lawns in temperate climate regions of North and South America and Europe [24]. The name “festuca” comes from the old Latin name “grass weed”. Fescues’ popularity is due to their high resistance to drought and low nitrogen demand; they do not require intensive care, while low sensitivity to lead allows their use nearby the communication routes [24]. Fescue grasslands do not contain other species characteristic for grassland habitats e.g., clover (*Trifolium* L. sp.), dandelion (*Taraxacum* F.H. Wigg. sp.), or daisies (*Bellis* L. sp.). This is because most of the fescues produce allelopathic compounds that inhibit the growth and development of neighboring plants. The use of fescue to reduce excess weeds in crops was intensively studied in the second half of the XX century. The water extracts of the dried shoots and roots of reed fescue (*Festuca arundinacea* Schreb.) inhibited the growth of black cabbage (*Brassica nigra* L.) [26] and negatively affected the seed germination, growth, and development of seedlings and the yield of trefoil (*Lotus corniculatus* L.) [27]. The application of an aqueous extract of fescue leaves resulted in the reduction of the yield of red clover (*Trifolium pratensis* L.) [28]. At the beginning of the 21st century, 78 species of fescue were examined, among which the seven most strongly limiting the weed infestation in field conditions were selected [25]. Further laboratory analyses have shown that the allelopathic potential of fescues corresponds to their root exudates. Fescues’ shoot extract inhibited the root growth of cress seedlings (*Lepidium sativum* L.) in 40%, while the root exudates limited the growth in 70% [25]. The main fescues’ root exudates component is *m*-Tyr, and the highest content of *m*-Tyr was recorded in the exudates of *F. rubra* spp. commutata and *F. rubra* spp. rubra [25]. The content of *m*-Tyr in the roots of fescue seedlings (about 6500 pmol mg^−1^ FW) was about 10 times higher than in leaves (590 pmol mg^−1^ FW). In dry seeds, *m*-Tyr was present at a much lower concentration, about 24 pmol mg^−1^ FW [29]. In fescues, epidermal cells of the root apex are responsible for the synthesis and release of exudates containing *m*-Tyr into the environment [25]. Fescues tolerate the presence of *m*-Tyr in the tissues possibly through its accumulation in intracellular or intercellular spaces [30]. The half-life of *m*-Tyr is rather short; it is estimated that, in filter paper bioassays, the *m*-Tyr half-life is less than 2 days and, in soil bioassays, less than 24 h [31].

Additionally, *m*-Tyr was detected in the donkey-tail spurge (*Euphorbia myrsinites* L.), but this plant does not exudate this NPAA into the environment [29]. As a secondary metabolite, *m*-Tyr is produced by some bacteria and may be a component of antibiotics, for example, pactamycin [32].

Contrary to the spontaneous formation under high ROS concentration, in fescues and donkey-tail spurge, *m*-Tyr is produced via enzymatic pathways by the transamination of *m*-hydroxyphenylpyruvate or the hydroxylation of Phe, respectively [33].

## 4. Mode of Action of *m*-Tyr

The direct mode of action linked to *m*-Tyr toxicity is its incorporation into the proteins, which alters physiological functionality [34]. In *Escherichia coli,* the incorporation occurs through the binding of *m*-Tyr to the tRNA^Phe^ [35]. As was shown for bacteria and human cells, the cytoplasmic or mitochondrial aminoacyl-tRNA synthetases are prone to catalyzing the binding of tRNA^Phe^ with *m*-Tyr [36]; thus, Tyr isomers at higher concentrations compete with Phe for tRNA^Phe^ ([37] and references herein).

The incorporation of *m*-Tyr into plant proteins was also demonstrated for 5-days-old arabidopsis seedlings [38]. The authors demonstrated that application of 10 μM *m*-Tyr reduced chlorophyll content, altered organellar biogenesis, and decreased photosynthesis and respiration rates.

As was mentioned, the toxicity of *m*-Tyr might be overcome by the Phe application. The recovery effect was described for tomato (*Solanum lycopersicum* L.) seedlings [39] and confirmed for arabidopsis [38]. The mechanism of the recovery effect is most probably based on the competition between Phe and *m*-Tyr [29]. The resistance to *m*-Tyr observed in arabidopsis mutants was linked to the accumulation of free Phe in leaves due to the overexpression of the genes encoding ADH, one of the enzymes of the Phe synthesis pathway [40].

In tomato seedlings, the mechanism of toxicity of *m*-Tyr is linked to altered ROS metabolism [39,41]. The treatment of tomato seedlings with *m*-Tyr led to the overproduction of ROS and the accumulation of carbonylated proteins ([39,41] and citations herein). Carbonyl groups in proteins are considered as markers of oxidative stress and ageing [42]. Similar to *m*-Tyr, the mode of action has been proven for another NPAA–canavanine (L-2-amino-4-guanidooxy-butanoic acid–CAN). This arginine analog, found in some Fabaceae plants, is considered as a toxic compound, protecting plants against herbivores ([43] and references herein). The direct mode of action of CAN is similar to *m*-Tyr; CAN is incorporated into proteins producing aberrant molecules [44]. Moreover, CAN supplementation into the growing medium of tomato seedlings stimulated ROS accumulation in roots and increased the level of carbonylated proteins [45]. Furthermore, *m*-Tyr decreased the enzymatic and non-enzymatic antioxidant activity in the roots of tomato seedlings [39] and decreased the generation of ONOO^−^ [46]. This indicates, that besides the alteration of the ROS metabolism, *m*-Tyr has an impact on the reactive nitrogen species (RNS) content [41,46]. In plants, the biological action of RNS is linked to post-translational protein modifications, including Tyr nitration. The formation of 3-nitro-tyrosine (3-NT) occurs through the addition of a nitro group (-NO_2_) in the *ortho* position of the Tyr ring via covalent binding [47]. Such modified, nitrated proteins lose their function [48,49]. There are no data indicating the possibility of the prevention of free *p*-Tyr nitration due to the *m*-Tyr formation. May *m*-Tyr incorporated into the protein structure also be nitrated? Does it physiologically matter? These questions need further investigation.

Duke [30] suggested that the possible mechanism of *m*-Tyr toxicity in plant cells is that *m*-Tyr could be converted into dihydroxyphenylalanine (L-DOPA), a compound of physiological meaning. The currently described action of *m*-Tyr in plants, observed after the application of synthetic DL *m*-Tyr, has been summarized in Table 1.

The inhibition of the growth of *Sacchromyces cerevisiae* after the addition of *m*-Tyr into the growing media was observed [35]. In *Escherichia coli*, *m*-Tyr altered a major part of the proteome as misfolded proteins crowded and formed aggregates [50]. To prevent this kind of damage, bacteria have a protective mechanism against mischarged AA. The activity of some tRNA synthetases is regulated by the quality control system that hydrolyzes the invalid AA before or after binding with the tRNA [50]. In *Escherichia coli* cells lacking the tRNA^Phe^ synthetase quality control, the exposure of *m*-Tyr led to protein aggregates accumulation. This was followed by the up-regulation of genes involved in the unfolded protein stress response [50].

The mode of action of *m*-Tyr in animals is mostly based on the incorporation of this NPAA into the proteins. This phenomenon was confirmed by Gurer-Orhan et al. [34] using radiolabeled *m*-Tyr added into the culture of hamster ovary cells. L-*m*-Tyr (0.2 mM) inhibited colony formation by 30% [34]. The simultaneous application of *m*-Tyr (0.2 mM) and Phe (1 mM) completely preserved the ability to proliferate. In humans, the elevated concentration of this Tyr isomer occurs in neurodegenerative diseases and diseases associated with oxidative stress and/or ageing: diabetes, artherosclerosis, and others ([18] and references herein) (Table 2). Moreover, *m*-Tyr can play a significant role in the cancer cells in animals. The concomitant tumor resistance is the phenomenon of the inhibition of secondary tumor implants or metastasis development in hosts that are already affected by the primary tumor [51]. Indeed, *m*-Tyr and *o*-Tyr were found to be factors leading to that resistance as they were discovered in the serum of tumor-bearing mice (*Mus musculus*). Administration of these NPAAs inhibited the growth of tumors in the murine models of cancer [52]. As was discussed, while secondary tumors are inhibited by circulating *m*-Tyr, the primary tumor microenvironment is protected by an accumulation of AA with counteracting properties (i.e. Phe) [53]. The primary tumor affected ROS generation, resulting in the increased *m*-Tyr and *o*-Tyr content [52]. This effect was observed in different human tumor types (prostate tumor, lung anaplastic, and nasopharyngeal carcinoma), where the application of *m*-Tyr led to the inhibition of cancer proliferation [54]. The treatment of cancer cells with *m*-Tyr induced the autophagy; however, the application of Phe reversed the toxic effect of *m*-Tyr on secondary cancer growth [52,54]. Studies on the key role of non-proteinogenic Tyr isomers in the mechanism of concomitant tumor resistance have shown an antiproliferative and anti-metastaic effect of *m*-Tyr [52,53,54]. These findings may be useful in the prevention of tumor metastasis. Moreover, *m*-Tyr can stop the growth of cells from tumor fragments that could have remained post-surgery. Importantly, for the therapeutic use of *m*-Tyr, no evidence of toxic effects of this NPAA at the concentration used for humans was found. After the oral application of 28 µmole *m*-Tyr kg^−1^ given to adults, the majority of the load was metabolized to 3-hydroxymandelic acid, 4-dihydroxyphenylacetic acid, and 3-hydroxyphenylacetic acid, detected in urine. In this experiment, no side effects have been observed [55]. Various examples of the *m*-Tyr mode of action in animals are presented in Table 2.

As was mentioned, exogeneous Phe might overcome the toxic effect of *m*-Tyr as the pool of free Phe in cells increases. An increased Phe level may be achieved by the inhibition of the activity of Phe hydroxylase (the enzyme responsible for Phe catabolism). On the other hand, the degradation of Tyr isomers may occur by the higher activity of Tyr-AT—the first enzyme in the Tyr catabolism pathway ([18] and citations herein). It was revealed that oxidative stress promotes the up-regulation of genes coding Tyr-AT in *Caenorhabditis elegans* [56]. The authors discussed that it might be the first step in the response against *m*-Tyr toxicity.

## 5. Summary

In animal cells, *m*-Tyr is formed as a product of Phe oxidation and thus is commonly accepted as a marker of the oxidative stress accompanying various pathological processes and diseases. In plants, besides Phe oxidation, *m*-Tyr is synthesized enzymatically, predominantly as a chemical weapon necessary for successful competition with neighbors in the environment. Despite the direct action of *m*-Tyr based on its incorporation into the protein structure, which is characteristic for both animal and plant tissues, there are several proofs that, in plants, *m*-Tyr alters the ROS and RNS metabolism [39,41].

Based on several data, the question as to why plants are more sensitive to *m*-Tyr than animals arose. According to previously published data, the Phe content in the leaves of arabidopsis is around 13.6 nmol g^−1^ FW [62], whereas the concentration of this AA in human skeletal muscles is about 2000-fold higher—around 46 µmol g^−1^ FW [63]. Such a difference in the Phe concentration may partially explain the *m*-Tyr toxicity in plants’ organs due to the limitation of the endogenous Phe necessary for the recovery effect. On the other hand, it may justify the existence of various enzymatic pathways of *m*-Tyr biosynthesis in plants.

## Figures and Tables

**Table 1:** Action of *m*-Tyr in various plants. Information on the methods/analytical procedure used in the experiments is given in parentheses.

Plant Material, *m*-Tyr Concentration	Effect of *m*-Tyr Application	References
Lettuce (*Lactuca sativa* L.) (DL-*m*-Tyr: 10–320 µM)	Inhibition of root growth (filter paper bioassays, soil bioassays)	[29,31]
Tomato (*Solanum lycopersicum* L.) (DL-*m*-Tyr: 50–250 µM)	Inhibition of root growth (filter paper bioassays) Accumulation of ROS (spectrophotometry) Increased emission of NO (fluorimetry) Modification of enzymatic and nonenzymatic cellular antioxidant system (spectrophotometry) Increased protein carbonylation level and protein nitration level (ELISA)	[39,46]
Arabidopsis (*Arabidopsis thaliana* ecotype Columbia (Col-0)) (DL-*m*-Tyr: 2.5–320 µM, according to personal information)	Inhibition of root growth and decreased leaves number (agar plate bioassays) Decreased total chlorophyll content (spectrophotometry), aberration in chloroplast ultrastructure (TEM), lowered photosynthesis rate (Clark-type electrode) Modification of mitochondria structure (TEM), lowered respiration rate (Clark-type electrode)	[38]

**Table 2:** Examples of pathophysiological effects related to the presence or application of *m*-Tyr in animal cells. Information on the methods/analytical procedure used in the experiments is given in parentheses.

Tissue/Biological Material	Level of *m*-Tyr	Physiological Effect Linked to *m*-Tyr and Accompanied Biochemical Changes	References
Human lenses	20.3 nmol g^−1^ protein	Cataract related with the lower content of soluble proteins (spectrophotometry) and Phe (HPLC)	[57]
Cerebrospinal fluid of newborn infants	20.5 nM	Hypoxic ischemic encephalopathy related with the increased content of ascorbic acid (HPLC), the higher *ortho*-Tyr/Phe and *m*-Tyr/Phe ratio (GC/MS), and the detection of non-protein-bound iron (spectrophotometry)	[58]
Human synovial fluid	0.5–3.5 µM	Rheumatoid arthritis. Determination of hydroxyradical attack on Phe (HPLC)	[59]
Human tumors (prostate, murine mammary carcinomas) in mice	* Injection of *m*-Tyr (67 mg kg^−1^ day^−1^)	Inhibition of the implantation of metastases (all treated and control mice were killed and metastases were counted)	[54]
T-lymphoma in BALB/c mice	* Injection of *m*-Tyr (0.2 mL of 500 μg mL^−1^ *m*-Tyr, daily for 5 days)	Decrease of tumor volume (histopathologic studies), the low expression of proliferation marker Ki-67 protein (immunostaining)	[52]
TF-1 erythroblast cell culture	* Treatment with *m*-Tyr (20 mg L^−1^)	Inhibition of cells proliferation (Bürker cell counting chamber) Induction of erythropoietin resistance (erythropoietin-response, cell counting)	[60,61]

* no information about used optical isomers.

## Data Availability

Not applicable.
